# Proteopeptidomic, Functional and Immunoreactivity Characterization of *Bothrops moojeni* Snake Venom: Influence of Snake Gender on Venom Composition

**DOI:** 10.3390/toxins10050177

**Published:** 2018-04-26

**Authors:** Fernanda Gobbi Amorim, Tassia Rafaela Costa, Dominique Baiwir, Edwin De Pauw, Loic Quinton, Suely Vilela Sampaio

**Affiliations:** 1 Laboratory of Toxinology, Department of Clinical Analysis, Toxicology and Food Sciences, School of Pharmaceutical Sciences of Ribeirão Preto, University of São Paulo—USP, Ribeirão Preto, SP 14040-903, Brazil; tassiarcosta@yahoo.com.br; 2 GIGA Proteomics Facility, GIGA Institute, University of Liège, 4000 Liège, Belgium; d.baiwir@uliege.be; 3 Laboratory of Mass Spectrometry, Department of Chemistry, University of Liège, 4000 Liège, Belgium; e.depauw@ulg.ac.be (E.D.P.); loic.quinton@ulg.ac.be (L.Q.)

**Keywords:** proteome, *Bothrops moojeni*, toxins, venomics

## Abstract

Venom composition varies across snakes from all taxonomic levels and is influenced by the snakes’ age, habitat, diet, and sexual dimorphism. The present study reports the first in-depth investigation of venom composition in male and female *Bothrops moojeni* (*B. moojeni*) snakes (BmooM and BmooF, respectively) through three proteomics approaches associated with functional, cytotoxic, and immunoreactivity characterization. Compared with BmooM venom, BmooF venom exhibited weaker hyaluronidase, metalloproteinase, and phospholipase activity; stronger recognition by anti-bothropic serum; 1.4-fold stronger cytotoxicity; and greater number of peptides. The increased L-amino acid oxidase expression probably accounted for the stronger immunoreactivity and cytotoxicity of BmooF venom. BmooF and BmooM venom shared only 19% peptides. Some venom components were gender-specific, such as phospholipases B, phospholipase inhibitor, and hyaluronidases in BmooM, and cysteine-rich secretory proteins in BmooF. In conclusion, we describe herein the first proteomics study of *B. moojeni* snake venom and an in-depth characterization of gender-specific differences in venom composition. Altogether, our findings not only stress the importance of considering the snake’s gender during antivenom production, but also help to identify new potential drugs and biotechnological tools.

## 1. Introduction

The term “venom” describes every type of toxic molecule produced by a specialized gland in animals. Venoms are deadly cocktails comprising a unique mixture of peptides and proteins naturally tailored by natural selection to act on vital systems of the prey or victim [1,2]. Despite the limited number of protein classes in venoms, their actions on the victim’s body may be much more complex due to their synergistic action that enhances their spread and pathophysiological effects [1].

Ophidic accidents are considered as a neglected disease and a serious health problem in most tropical countries [3]. In Brazil, snakes belonging to the genus *Bothrops* (Viperidae family, Crotalinae subfamily) caused 85% of the ophidic accidents reported [4,5]. Bothropic accidents are characterized by intense local inflammation associated with pain, myonecrosis, oedema, and haemorrhage [6]. The species *Bothrops moojeni*, popularly known as “caiçara” or “jararacão”, is found in midwestern and southeastern Brazil [7].

Venoms can be considered as natural libraries of largely unexplored bioactive compounds containing many promising candidates for a range of medical applications [8]. The application of omics technologies, such as proteomics and transcriptomics, has enabled significant progress in the understanding of animal venom complexity. A new term to refer to these in-depth studies emerged in the last decade, venomics [1,9]. Although experimental approaches have helped to achieve a more comprehensive knowledge of venom composition, these toxins are still considered as extremely complex mixtures whose composition can be influenced by several factors [10]. 

Variation in venom composition is an ubiquitous phenomenon in snakes at all taxonomic levels, from temporal variation within individuals to higher levels [11]. The most well-studied snake species are from the genus *Bothrops* is *Bothrops jararaca* (*B. jararaca*), whose venom composition changes due to intraspecific and geographical variability [12,13,14], as well as due to ontogenetic variability such as diet [15,16,17,18], age [16,19] and sexual dimorphism [20,21,22,23,24].

The snake’s age and sexual dimorphism seem to influence the incidence of snakebites from the *Bothrops* genus. Female and juvenile snakes have caused the majority of the ophidic accidents treated at the Vital Brazil Hospital (São Paulo, SP, Brazil) [25]. Venom from female *B. jararaca* displays stronger hyaluronidase, haemorrhagic, and lethal activity [6]. Large *B. moojeni* snakes have caused most snakebites in the region of São José do Rio Preto (State of São Paulo, Brazil); their victims have intense local reactions of oedema, necrosis, and secondary infection, and a slight loss of coagulant function [26]. Compared with snakebites caused by *B. jararaca*, ophidic accidents caused by *B. moojeni* are associated with higher incidence of non-clotting blood and local swelling/necrosis [27].

Scientists from all over the world have used proteomics approaches to characterize venoms and broaden the knowledge about these toxic cocktails. Recent advances in instrumentation have enabled sensitive and comprehensive shotgun protein analysis in a high-throughput manner [10]. Shotgun proteomics-based approaches have opened new perspectives to overcome the frontiers of venom biology by enabling the identification of less abundant proteins and post-translational modifications, and providing relative and absolute protein quantitation [28].

Although the proteomics profile of venoms from more than 200 snake species has already been described [10], there are no reports on proteomics studies with *B. moojeni* snake venom. Our research team has recently reported the transcriptomics analysis of *B. moojeni* snake venom gland [29]. To date, there are no studies that examined how sexual dimorphism can affect *B. moojeni* snake venom composition. In this sense, the present study represents the first in-depth investigation of *B. moojeni* snake venom composition through proteomics approaches associated with functional, cytotoxic, and immunoreactivity characterization.

## 2. Results and Discussion

### 2.1. Functional and Cytotoxic Characterization

Sexual dimorphism is evident in *Bothrops moojeni*, in which female specimens are larger than male specimens, with a median body size of 1034 mm (range 760–1330 mm) and 827 mm (range 590–1060 mm), respectively [7]. The larger body size of females snakes may result from size-dependent fecundity to provide more space for reproductive organs and embryos within the body cavity [30]. The influence of sexual dimorphism on venom composition is well-documented in the literature [22,24,31]. *Bothrops jararaca* is the most well-studied *Bothrops* species with respect to gender influence on venom composition [20,21,22,23,32]. To the best of our knowledge, this is the first report on gender-based variation in the *B. moojeni* venom composition.

**Figure 1:**
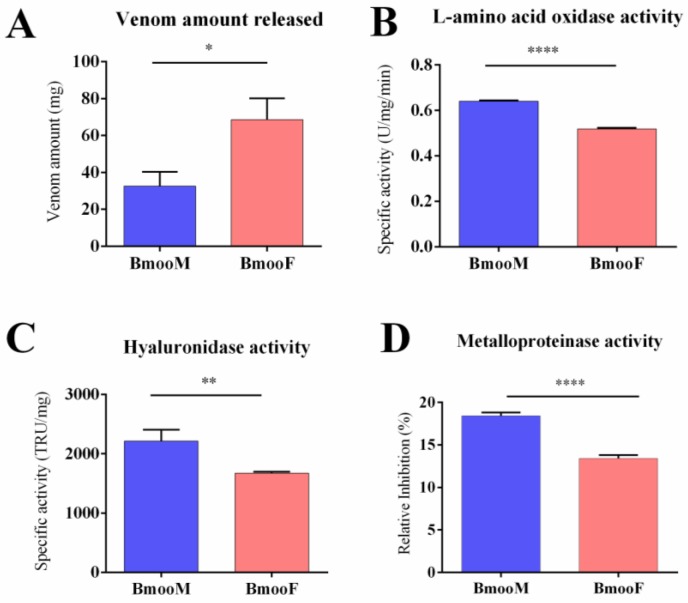

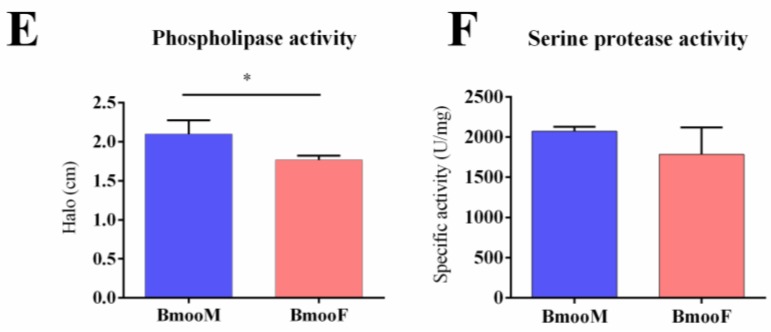
Functional characterization of *B. moojeni* venom stratified by snake gender. BmooF = female snake; BmooM = male snake. (**A**) Amount of venom released during manual extractions; (**B**) L-amino acid oxidase specific activity determined using *o*-phenylenediamine as substrate; (**C**) Hyaluronidase specific activity determined by hydrolysis of hyaluronan, measured through a turbidimetric assay. TRU: turbidity-reducing units; (**D**) Metalloproteinase activity determined using the azocasein assay. Results are expressed as relative inhibition of the activity after venom incubation with ethylenediamine tetraacetic acid (EDTA); (**E**) Phospholipase activity determined using egg-yolk agar plate medium. The size of the halos formed indicate the rate of phospholipase activity; (**F**) Serine protease specific activity determined using Nα-*p*-tosyl-L-arginine methyl ester (TAME) as substrate. The results are expressed as mean ± SD. *Statistics* (Student’s *t* test): (**A**) * *p* < 0.011; (**B**) **** *p* < 0.0001; (**C**) ** *p* < 0.0079; (**D**) **** *p* < 0.0001; (**E**) * *p* < 0.0341; and (**F**) *p* = 0.2145.

In the present study, we performed several analyses to characterize the venom composition in male and female *B. moojeni* snakes (BmooM and BmooF, respectively). The snake genders significantly differed with respect to the amount of venom released (*p* = 0.011): BmooF released a two-fold greater amount of venom than BmooM (68.60 mg ± 11.60 mg and 32.60 mg ± 7.77, respectively) (Figure 1A). The venom amount released varies according to several factors, including the aim of the bite (defence or predation), snake species, and snake size [33,34,35]. As the *B. moojeni* specimens studied herein were housed in the same serpentarium, received the same feeding, and underwent the same procedure of venom extraction, we can conclude that their body size directly influenced the amount of venom released. It is worth noting, that female *B. jararaca* snakes produce five-fold greater amounts of venom than male snakes from the same species [23].

We examined the activity of five enzyme classes in BmooM and BmooF venom samples (Figure 1B–F). The specific activity of four enzyme classes—L-amino acid oxidase (LAAO), hyaluronidase, metalloproteinase, and phospholipase—was significantly higher in BmooM than in BmooF; only the serine protease activity did not differ between the snake genders. A previous study with *B. jararaca* has reported that (i) venom from female snakes exhibits stronger hyaluronidasic and haemorrhagic effect; (ii) venom from male snakes exhibits stronger coagulant, phospholipase, and myotoxic activity; and (iii) venom from both snake genders displays similar proteolytic and oedematogenic activity [23]. Other studies have detected stronger proteolytic activity in venom from female *B. jararaca* snakes [21,22].

**Figure 2:**
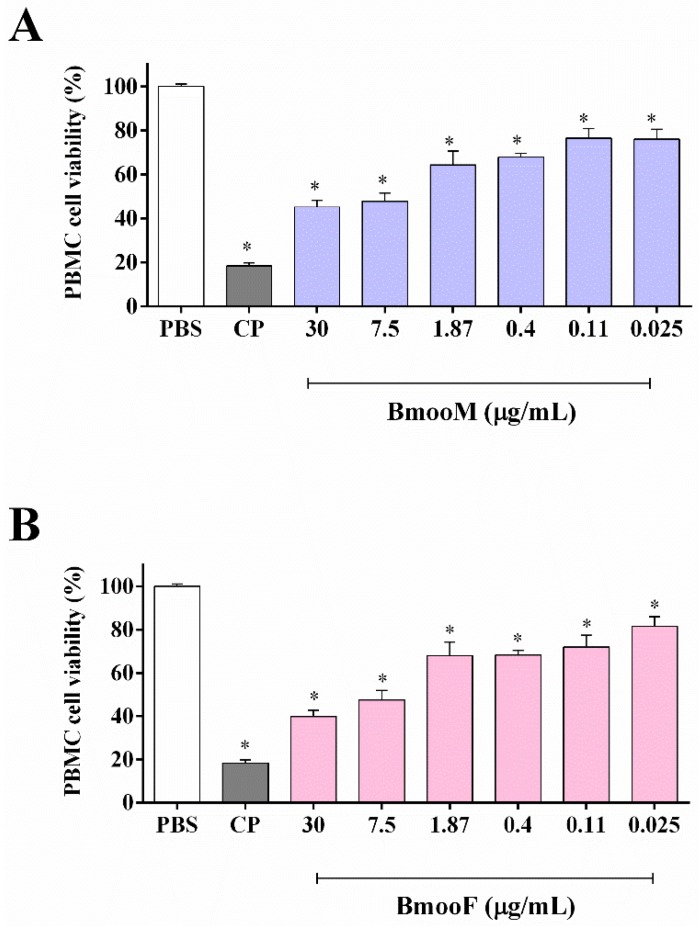
Cytotoxicity of *B. moojeni* venom from male (BmooM, panel (**A**)) and female (BmooF, panel (**B**)) snakes towards peripheral blood mononuclear cells (PBMC). The cell viability was assessed by the MTT assay after 24 h of treatment with venom. CP: cisplatin at 33.0 μg/mL (positive control). PBS: phosphate buffered saline solution (negative control). Results are expressed as mean ± SD of three independent experiments (*n* = 3). The IC_50_ values were 2.41 μg/mL (BmooF) and 3.35 μg/mL (BmooM). *Statistics*: * *p* < 0.05 versus PBS (one-way ANOVA followed by the Tukey’s test).

Next, we examined the cytotoxic potential of *B. moojeni* venom towards peripheral blood mononuclear cells (PBMC). In the range of concentrations tested (0.025–30 μg/mL), BmooM and BmooF venom significantly decreased PBMC cell viability (*p* < 0.05) (Figure 2) in a concentration-dependent manner, affording IC_50_ values of 2.41 and 3.35 μg/mL, respectively. It means that BmooF was 1.39 times more cytotoxic than BmooM towards PBMC. In line with our finding, the literature reports that *B. jararaca* venom from female snakes is eight-fold more lethal to Swiss mice than venom from male snakes [23].

### 2.2. Immunoreactivity Characterization

**Figure 3:**
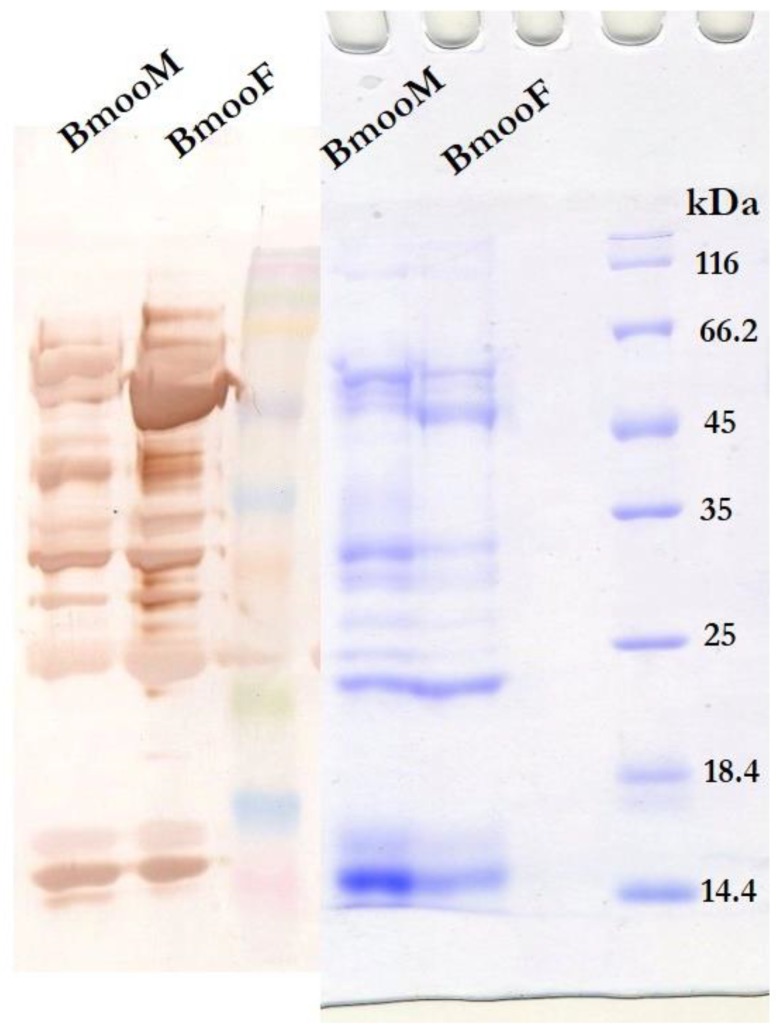
Recognition of venom components from male (BmooM) and female (BmooF) *B. moojeni* snakes by anti-bothropic serum. Western blot of venom samples (20 μg) using anti-bothropic serum and anti-horse IgG antibodies. After SDS-PAGE separation on 15% polyacrylamide gel under reducing conditions (**Right**), the protein bands were transferred to a nitrocellulose membrane and immunostained with the indicated antibodies (**Left**).

To characterize the immunoreactivity of BmooM and BmooF venom, we analysed the profile of venom proteins recognized by anti-bothropic serum using Western blotting and enzyme-linked immunosorbent assay (ELISA). The serum recognized more protein bands in BmooF than in BmooM, even though we used the same venom amount and antibody dilution for both genders in the Western blotting assay (Figure 3). The ELISA assay confirmed the recognition of a significantly greater amount of proteins in BmooF. BmooF showed 99.57% (SD ± 0.50) of recognition by the anti-bothropic serum compared to BmooM with 93.32% (SD ± 0.71) (*p* < 0.05). In addition, the serum strongly recognized a protein band between 45 and 66.2 kDa. Here we report for the first time, that snake sexual dimorphism influences the immunoreactivity of *B. moojeni* venom, and that BmooF venom is more antigenic than BmooM venom.

### 2.3. Proteomic Analyses

#### 2.3.1. Fractionation by Reversed-Phase High-Performance Liquid Chromatography (HPLC) and Matrix Assisted Laser Desorption Ionization-Time of Flight (MALDI-TOF) Mass Spectrometry

**Figure 4:**
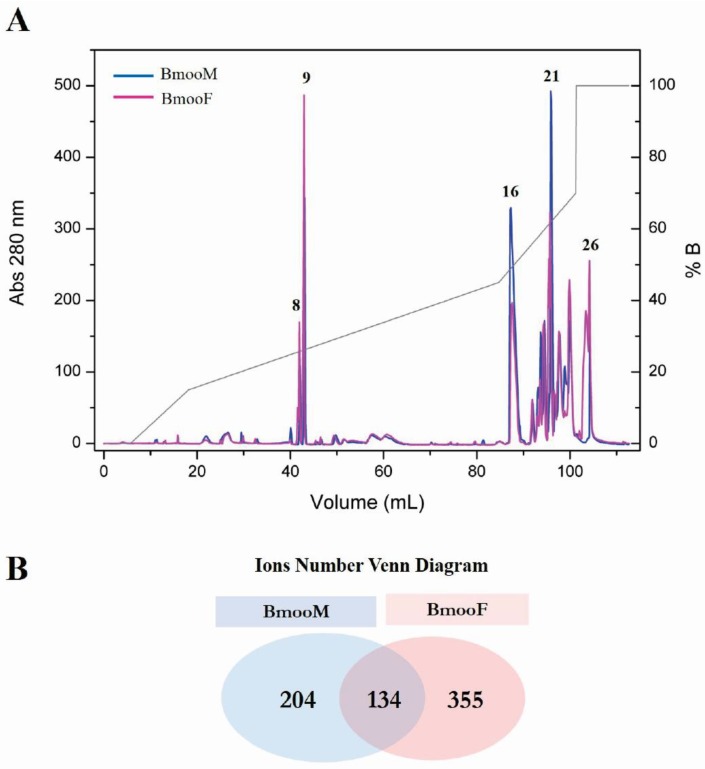
Analysis of venom composition in male (BmooM) and female (BmooF) *B. moojeni* snakes using HPLC coupled to MALDI-TOF mass spectrometry. (**A**) Comparative chromatographic profile of BmooM and BmooF venom obtained after reversed-phase HPLC analysis using a linear concentration gradient of 70% acetonitrile (solvent A) and 0.1% trifluoroacetic acid (solvent B), at a flow rate of 1 mL/min. The numbers represent the elution time (in minutes) of five fractions whose peak intensity and/or retention time differed considerably between the snake genders; (**B**) Venn diagram of the number of ions obtained by MALDI-TOF mass spectrometry analysis of the five fractions selected in (**A**).

The reversed-phase HPLC analysis of 5 mg of crude BmooM and BmooF venom samples, according to Bernardes et al. (2013) protocol, resulted in 27 fractions. Comparison between the chromatographic profiles of BmooM and BmooF evidenced that they differed with respect to the peak intensity and/or retention time of some fractions (Figure 4A). Five fractions that differed considerably between the snake genders were analysed by MALDI-TOF mass spectrometry in order to obtain an overview of their components. BmooF venom presented more peptides (51%) than BmooM venom (29%), and venom from both genders shared only 19% peptides (Figure 4B). 

#### 2.3.2. Analysis in the AmaZon Speed electron transfer dissociation (ETD) Ion Trap Mass Spectrometer

**Figure 5:**
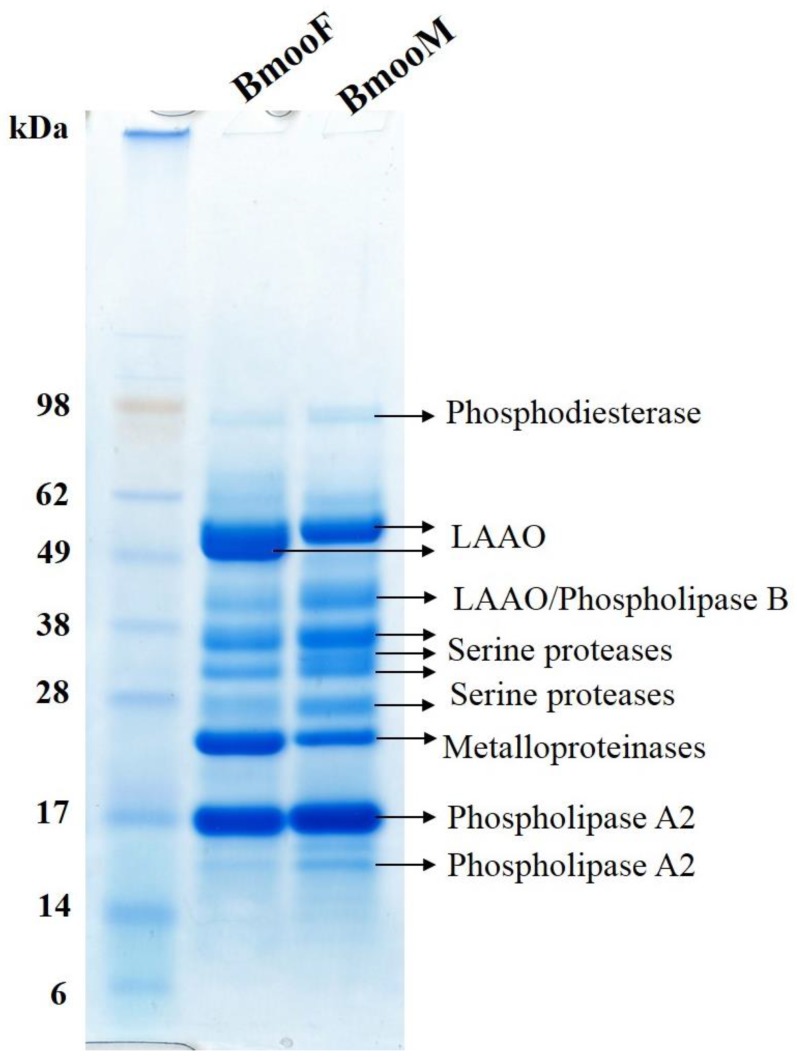
Identification of venom components from male (BmooM) and female (BmooF) *B. moojeni* snakes. The protein bands separated by SDS-PAGE were digested and the resulting tryptic peptides were sequenced using the AmaZon Speed ETD Ion Trap mass spectrometer and further analysed against UniProt database.

To better understand the differences in venom composition between the snake genders revealed by the Western blotting assay, we performed digestion of each electrophoretic band using an automatized robot and analysed the samples using the AmaZon Speed ETD Ion Trap mass spectrometer (Bruker Daltonics, Billerica, MA, USA). For data analysis, we used the database created for “Snake Venom” downloaded from UniProt in June 2017. We found that the venom component was a function of its electrophoretic band (Figure 5). It is worth noting that BmooF venom presented an intense electrophoretic band between 49 and 62 kDa recognized by anti-bothropic serum in the Western blotting assay (Figure 3); this band was identified as LAAO (Figure 5). BmooF venom had a strong metalloproteinase band near 28 kDa, while BmooM venom had several bands identified as serine proteases, corroborating the functional data reported in Figure 1. Although BmooF presented an intense electrophoretic band on SDS-PAGE related do LAAO, this enzyme group was shown to be less active in BmooF compared to BmooM. LAAOs are enantioselective flavoenzymes that catalyze the stereospecific oxidative deamination of L-amino acids. These enzymes present an unstable enzyme activity, as it was already reported in the literature [36,37]. The mechanism that affects the enzyme activity of LAAOs in the venom are complex. However, it may be related with oxidation, pH and temperature changes, or non-protein inhibitors that may be present in the snake venom, which may cause alterations in the microenvironment of the flavin adenine dinucleotide (FAD) cofactor [36,37]. However, the activity of this enzyme does not seem to influence the cytotoxicity and immunoreactivity of BmooF. 

#### 2.3.3. Shotgun Proteomics on Q-Exactive Orbitrap Mass Spectrometer

Proteomic analysis of BmooM and BmooF venom composition was performed on a Q-Exactive Orbitrap mass spectrometer. The venom samples were digested as described in the previous Section and analysed by mass spectrometry. The raw data obtained were analysed using Peaks Studio v. 7.0 software (Waterloo, ON, Canada, 2007) considering (i) the transcripts from *B. moojeni* transcriptome deposited by Amorim et al. (2017), and (ii) a database created for “Snake Venom” downloaded from UniProt in January 2018 containing 33,138 protein and peptide sequences and the transcriptome data. We analysed the data considering the SPIDER search in order to have the de novo sequencing results.

##### Analysis Against the Transcriptome Database

Pooled venom samples were analysed against the transcriptome database. De novo analysis (PEAKS) resulted in 9206 Peptide Spectrum Matches, and 2011 peptides that represented 92 identified proteins using the *B. moojeni* transcriptome. Among the transcripts related to venom components identified in the transcriptome, 5.84% were found in the proteome of *B. moojeni* snake venom. In the set of proteins identified (Supplementary Materials, Table S1), 67 presented more than two unique peptides and 22 presented two unique peptides. Nine proteins (Table 1) out of the 33 new full-length venom components described by Amorim et al. (2017) were identified in the proteome, confirming that these transcripts are expressed in the venom.

##### Analysis Against Uniprot Database

Mass spectrometry analyses resulted in almost the same number of MS and MS/MS scans for both snake genders. After applying the parameters described in the Methods section, BmooM venom presented more Peptide Spectrum Matches than BmooF venom, but the latter presented more peptide sequences that matched the database information; hence, a greater number of proteins were identified in BmooF venom (Table 2).

**Figure 6:**
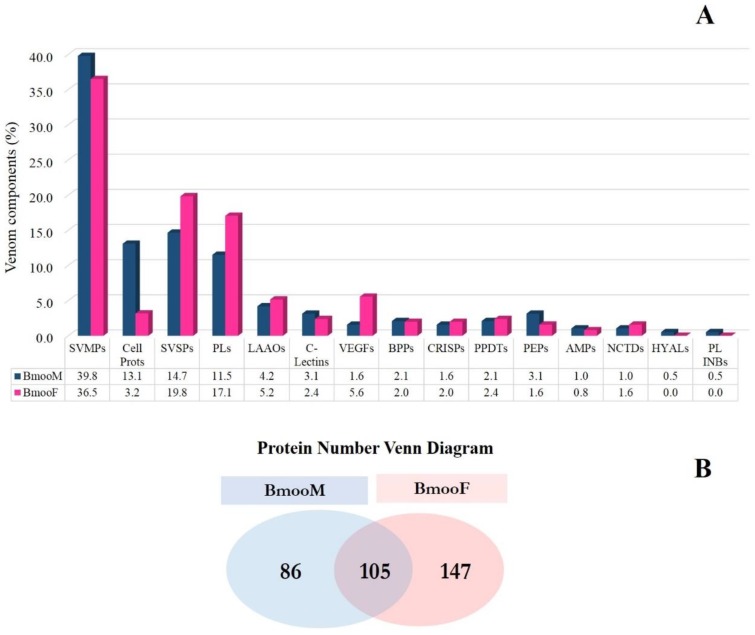
Distribution of protein classes in venom from male (BmooM) and female (BmooF) *B. moojeni* snakes determined by shotgun-proteomics. (**A**) Relative distribution of protein classes in BmooM and BmooF venom. AMPs: antimicrobial peptides, BPPs: bradykinin-potentiating peptides, Cell Prots: cellular proteins, C-Lectins: C-type lectins, CRISPs: cysteine-rich secretory proteins, HYALs: hyaluronidases, LAAOs: L-amino acid oxidases, NCTDs: nucleotidases, PEPs: peptidases, PLs: phospholipases, PL INBs: phospholipase inhibitors, PPDTs: phosphodiesterases, SVMPs: snake venom metalloproteinases, SVSPs: snake venom serine proteases, VEGFs: vascular endothelial growth factors; (**B**) Venn diagram with the proteins identified by mass spectrometry.

BmooM and BmooF venom presented 1860 and 2061 peptides, respectively, and shared 1288 peptide sequences. These results reflected the number of proteins and protein groups identified; 191 and 252 proteins from BmooM and BmooF venom matched the database, respectively (Figure 6), corroborating the findings of chromatographic fractionation and MALDI-TOF mass spectrometry (Figure 4).

In this study, we identified 15 distinct classes of venom components; all the proteins related to non-venom components were grouped in the “Cellular proteins” group. Comparative analysis of the protein classes identified using this proteomics approach revealed that they were almost similarly distributed between BmooM and BmooF venoms, and only some classes predominated in one snake gender (Figure 6A). BmooM venom exhibited a higher proportion of metalloproteinases, which may explain the results from the azocasein assay depicted in Figure 1—C-type lectins, hyaluronidases, peptidases, and phospholipase inhibitors. BmooF venom contained greater amounts of serine proteases, phospholipases, LAAOs, vascular endothelial growth factors, cysteine-rich secretory proteins (CRISPs), and nucleotidases. The increased levels of LAAO identified here corroborated data from cytotoxicity (Figure 2B) and Western blotting (Figure 3) assays. Venom from both snake genders exhibited similar levels of bradykinin-potentiating peptides, phosphodiesterases, and antimicrobial peptides.

BmooM and BmooF venoms shared 31% of proteins and had 26% and 44% of exclusive proteins, respectively (Figure 6B). The full list of peptides found is reported in Supplementary Materials, Table S2. All the proteins identified exclusively in venom from each snake gender are listed in Supplementary Materials, Tables S3 and S4. 

Two phospholipases B (coverage: 34.5% and 22.7%), one phospholipase inhibitor (coverage: 12.38%), and one hyaluronidase (coverage: 21.8%) were found exclusively in BmooM (Supplementary Materials, Table S3). Phospholipases catalyse the release of fatty acids and lysophospholipids from membrane phospholipids by acting on four different sites of the substrate. Phospholipase B cleaves phospholipids at sn-1 and sn-2 positions [38]. There are few reports on detection of phospholipases B in snake venoms, which is considered a minor snake venom protein [39]. This is the first report on the expression of phospholipase B in *B. moojeni* venom, which corroborates our previous report about its expression in the transcriptome of *B. moojeni* venom gland [29].

A phospholipase inhibitor that was exclusively identified in BmooM venom shared identity with phospholipase A2 inhibitor subunit gamma B-like of *Crotalus adamateus*. Phospholipase inhibitors were already identified in *B. moojeni* venom. BmjMIP, a phospholipase A2 myotoxin inhibitor protein from *B. moojeni* snake plasma, suppresses a variety of damaging effects of basic and acidic phospholipases A2 from *Bothrops* venoms, including myotoxicity, oedema induction, cytotoxicity, bactericidal, and lethal [40]. This is the first report of a phospholipase inhibitor in the venom of *B. moojeni* snake.

The hyaluronidase exclusively found in BmooM venom shared similarity with the enzyme from *Agkistrodon contortrix* venom. This finding confirms the previous report on the presence of hyaluronidase in the transcriptome of *B. moojeni* snake venom gland [29], and may explain the higher hyaluronidase activity of BmooM venom.

BmooF venom contained two exclusive cysteine-rich secretory proteins (CRISPs) (coverage: 28.3% and 26.6%) (Supplementary Materials Table S4), corroborating the findings from the transcriptome of *B. moojeni* snake venom gland [29]. CRISPs exist in venom from several snake species, but their biological activities have not been fully understood. They can exert neurotoxicity by blocking several ion channels [41]. Together, our results demonstrated that the composition of *B. moojeni* venom varied according to the snake gender. 

## 3. Discussion

Mass spectrometry has emerged as a tool for large-scale protein analysis. Over the last decade, the resolution, mass accuracy, sensitivity, and scan rate of mass spectrometers used to analyse proteins have greatly improved, and the introduction of hybrid mass analysers has empowered proteomic analysis. One way to perform proteomics studies is the use of the “bottom-up” approach, which refers to the characterization of a given protein by analysing the peptides produced after its digestion by a protease [42]. 

Shotgun proteomics, i.e., the analysis of a mixture of proteins, enables the mass spectrometry analysis of peptides from complex samples that underwent tryptic digestion. In this methodology, complex mixtures of peptides are separated by liquid chromatography and immediately analysed in a mass spectrometer coupled to the chromatograph (LC-MS/MS) [42,43]. The use of shotgun proteomics to carry out a wide range of research experiments has promoted advances in biological discoveries. As this approach enables analysis of complex protein mixtures and the fast generation of a global profile of proteins within a mixture, it has been intensively applied to proteome profiling, protein quantitation, and analysis of post-translational modifications and protein-protein interactions [28].

The application of shotgun proteomics in venomics fields provides several advantages, such as (i) high sample-to-sample reproducibility, which affords comparison on an equal basis of many venom samples with respect to the animal gender, age, and environmental differences as well as analysis of how strongly such differences affect the venom composition; (ii) the high sensitivity and dynamic range of equipment setups enable identification of over 4000 proteins from a complex mixture in a single run, and of the proteome profile of an organism within one hour; (iii) minimization of the background coming from human keratins and trypsin when compared to in-gel protocols, due to reduced sample manipulation; and (iv) compatibility with peptide fractionation/enrichment and quantification methods [28,44,45].

Although the application of omics approaches in toxinology has enabled the in-depth elucidation of venom components from the *B. moojeni* venom gland, it remains unclear how the transcripts are processed into mature proteins. The correlation between mRNA and protein levels is well-documented and has been attributed to differences in translational efficacy, codon usage/bias, and mRNA versus protein stability [46]. This fact may explain why some transcripts detected in the full-length sequences of the transcriptome of *B. moojeni* venom gland [29] were not identified in the present proteomics study. However, both studies found similar patterns of venom composition, especially for metalloproteinases, serine proteases, and phospholipases.

The integration of proteomics approaches with functional, cytotoxic, and immunoreactivity characterization in the present study allowed us to understand how sexual dimorphism affects *B. moojeni* venom composition. Venom from male snakes presented stronger metalloproteinase activity, which was confirmed by the increased expression of this enzyme class in the proteome. Female snakes produced larger venom amounts, with a wider variety of components and stronger cytotoxicity and antigenic potential. In line with these findings, venom from female snakes exhibited higher expression levels of some enzymes that may contribute to its cytotoxicity and immunoreactivity, such as LAAOs. In addition, proteomics analysis enabled identification of venom components that were expressed exclusively in each *B. moojeni* snake gender; male snakes exclusively expressed phospholipases B, phospholipase inhibitor, and hyaluronidase, while female snakes exclusively expressed CRISPs. These findings stress the importance of considering the intraspecific differences during antivenom production and searching for new potentially bioactive molecules.

In summary, the present study described the results of the first proteome from *B. moojeni* snake venom, associated with the functional, cytotoxic, and immunoreactivity characterization and fractionation of venom from male and female snakes, with the purpose of analyzing the influence of sexual dimorphism in venom composition. Several studies have already reported the differences among venom components in other species belonging to the genus *Bothrops*. Although *B. jararaca* is a well-studied species, it is important to extend the research to other species from the *Bothrops* genus in order to unravel how intra- and interspecific variations affect the venom composition, and to perform an in-depth characterization of this complex mixture. Knowledge about interspecific and intersexual differences in venom composition may help to produce more specific antivenoms, understand the symptoms of ophidic accidents, and identify new molecules that can be potential drugs and biotechnological tools.

## 4. Methods

### 4.1. Chemicals and Materials

Azocasein, horseradish peroxidase, hyaluronan, hydrogen peroxide, l-leucine, Nα-*p*-tosyl-l-arginine methyl ester, *o*-phenylenediamine, peroxidase-labelled anti-horse IgG antibody, phosphate buffered saline with Tween, and Sigma FAST™ 3,3’-diaminobenzidine tablets were purchased from Sigma-Aldrich (St. Louis, MO, USA). *Coomassie* Brilliant *Blue* G-250 and trypsin were obtained from Thermo Scientific *Pierce* (Waltham, MA, USA). NuPAGE MES gel and SeeBlue Plus2 Pre-Stained standard were acquired from Invitrogen (Darmstadt, Germany).

The following chemicals and materials were purchased from different suppliers: anti-bothropic serum (Fundação Ezequiel Dias, Belo Horizonte, MG, Brazil), cisplatin (Incel^®^, Darrow, Rio de Janeiro, RJ, Brazil), Peptide Calibration Standard II™ (Bruker Daltonics, Bremen, Germany), PVDF membrane (Invitrolon™ PVDF/Filter Paper Sandwiches; Invitrogen™, Carlsbad, CA, USA), trichloroacetic acid (Thermo Fisher Scientific, Waltham, MA, USA), and ZipTip^®^ pipette tips with C18 reversed-phase resin (Millipore, Darmstadt, Germany).

### 4.2. Venoms

Venoms from male and female specimens of *B. moojeni* were extracted separately (at least three manual extractions of each snake specimen), pooled, and named as BmooF (female venoms) and BmooM (male venoms). The animals were captured in the region of Ribeirão Preto, SP, Brazil, and housed in the serpentarium of Ribeirão Preto Medical School at the University of São Paulo (FMRP-USP, Ribeirão Preto, SP, Brazil), in compliance with the guidelines of Ibama (Brazilian Institute of Environment). The venom samples were dried and stored at −20 °C until use.

### 4.3. Functional Characterization

#### 4.3.1. L-Amino Acid Oxidase (LAAO) Activity

LAAO activity of the BmooM and BmooF venom samples was determined using the method reported by Bordon and collaborators [36], with modifications. Briefly, 2 μg of venom were incubated for 30 min, at room temperature, with 2 mM *o*-phenylenediamine, 1 U/mL horseradish peroxidase, 5 μM l-leucine, and 1 M Tris-HCl buffer pH 7.2. The reaction was stopped with 500 μL of 10% citric acid and absorbance was recorded at 490 nm. The absorbance values were used to calculate the LAAO specific activity in U/mg/min, which is the amount of H_2_O_2_ (μmol) formed per minute per mg of protein. The amount of H_2_O_2_ formed was quantified from a standard curve of H_2_O_2_ concentration expressed in nmol/min.

#### 4.3.2. Hyaluronidase Activity

Hyaluronidase activity of the BmooM and BmooF venom samples was determined by a turbidimetric assay adapted to 96-well microplates [47], under the best enzyme conditions established for snake’s hyaluronidase [48]. The venom samples were added to 100 μL of 0.2 M sodium acetate buffer pH 5.5 supplemented with 0.15 M NaCl and 10 μg of hyaluronan (0.5 mg/mL in water), and further incubated for 1 h, at 37 °C. The unhydrolyzed hyaluronan was precipitated with 100 μL of 5% cetyltrimethylammonium bromide dissolved in 4% NaOH. The turbidity was monitored at 400 nm in a microplate reader (Sunrise, Tecan, Männedorf, Switzerland). The calibration curve was constructed with 0–10 μg of hyaluronan. Turbidity-reducing units (TRU) are expressed as the amount of enzyme required to hydrolyse 50% (5 μg) of hyaluronan; the specific activity is expressed as turbidity-reducing units per milligrams of enzyme (TRU/mg).

#### 4.3.3. Metalloproteinase Activity

The proteolytic activity of BmooM and BmooF venom samples was quantified by the method of Wang and collaborators [49], with modifications. First, 19 μg of each venom sample was incubated with 5 μL of 100 mM ethylenediamine tetraacetic acid (EDTA) for 15 min, at 37 °C. Then, 5 μL of 50 mM Tris-HCl pH 8.8 and 85 μL of an azocasein solution (5 mg/mL in 50 mM Tris-HCl buffer, pH 8.8) were added to the samples. After a 90-min incubation at 37 °C, the reaction was stopped by adding 5% trichloroacetic acid to the reaction mixture and centrifuging it (1000× *g*, 5 min). Aliquots of the supernatant (150 μL) were transferred to a 96-well microplate and mixed with an equal volume of 5 mM NaOH. The absorbance was recorded at 450 nm. One unit of proteolytic activity corresponds to an increase of 0.01 unit of absorbance at 450 nm, and the specific activity was calculated in units per milligram of protein (U/mg). The metalloproteinase activity was calculated considering the relative difference between the specific activity before and after inhibition with EDTA.

#### 4.3.4. Phospholipase Activity

Phospholipase activity of BmooM and BmooF venom samples was assessed in Petri dishes, as described by Gutiérrez et al. [50], with the following modifications: agarose was replaced by agar, and erythrocytes were not used. Briefly, a gel containing 0.01 M CaCl_2_, egg yolk diluted in phosphate-buffered saline (PBS) at pH 7.2 in the ratio 1:3 (*v*/*v*), 1% bacteriological agar, and 0.005% sodium azide was prepared in Petri dishes. Then, 40 μL of the samples were applied into 5-mm diameter holes made in the gel, followed by incubation at 37 °C overnight. The formation of translucent halos around the holes in the gel indicated phospholipase activity. The halo diameter was measured in millimeters. 

#### 4.3.5. Serine Protease Activity

The serine protease activity of the BmooM and BmooF venom samples was tested using the substrate Nα-*p*-tosyl-l-arginine methyl ester (TAME), according to the method of Hummel [51], with modifications. The reaction mixture consisted of 0.2 mL of venom (7 µg; prepared in deionized water), 0.3 mL of 0.01 M TAME (final concentration of 1 mM), and 2.5 mL of buffer solution (0.02 M Tris-HCl and 0.15 M KCl pH 8.1). After a 30-min incubation at 37 °C, the absorbance was recorded at 247 nm. One TAME unit represents an increase of 0.01 absorbance units resulting from substrate hydrolysis by the enzyme. The results were expressed as specific activity, related to TAME units per milligram of protein (U/mg).

### 4.4. Cytotoxicity Assay

Cytotoxicity of BmooM and BmooF venom samples towards PBMC was assessed by the MTT assay [52]. PBMC were treated for 24 h with different venom concentrations (0.025, 0.11, 0.4, 1.87, 7.5, and 30 μg/mL), PBS (negative control) or 33.0 μg/mL of cisplatin (positive control). The cisplatin concentration selected theoretically inhibits 50% of the PBMC cell growth (IC_50_) [53].

At *t* = 24 h, MTT solution was added to the wells (500 μg/mL, final concentration). After a 4-h incubation, the reaction was stopped by adding 100 μL of dimethyl sulfoxide (DMSO) to the wells. The percentage of PBMC cell viability after treatment with each venom concentration were used to calculate the IC_50_ value, using the CalcuSyn 2.1 software. The assay was performed in biological and experimental triplicate. The Human Research Ethics Committee from the School of Pharmaceutical Sciences of Ribeirão Preto, University of São Paulo (Ribeirão Preto, SP, Brazil) approved the study protocol (CEP-FCFRP/USP protocol n. 334). All the procedures conducted in humans complied with Resolution 466/12 of the Brazilian National Health Council, which follows the recommendations of the 1964 Helsinki declaration and its later amendments.

### 4.5. Immunoreactivity Characterization

#### 4.5.1. Western Blotting

First, 20 µg of BmooM and BmooF venom samples were subjected to 15% polyacrylamide gel electrophoresis (SDS-PAGE) according to the protocol reported by Laemmli [54]. The samples were dissolved in appropriate buffer (0.5 M Tris-HCl buffer, pH 6.8, plus 10% SDS, 10% 2-β-mercaptoethanol, and 0.5% bromophenol blue dye), boiled at 100 °C, loaded on 15% polyacrylamide gel, and ran at 150 V. Second, protein bands were transferred to a PVDF membrane through electroblotting at 200 V for 60 min. The membrane was blocked with 5% skim milk in TBS buffer (50 mM Tris-HCl, pH 8.0, 150 mM NaCl) for 60 min at room temperature and under mild agitation. Then, the membrane was washed with TBS buffer and incubated for 1.5 h with anti-bothropic serum diluted 1:3000 in TBS. After four washes with TBS, the membrane was incubated with peroxidase-labelled anti-horse IgG antibodies diluted at a ratio of 1:5000 for 1.5 h. The venom bands recognized by the anti-bothropic serum were revealed using Sigma FAST™ 3,3’-diaminobenzidine tablets in 5 mL of TBS. The membrane was scanned after drying. 

#### 4.5.2. Enzyme-Linked Immunosorbent Assay (ELISA)

Microplate wells were sensitized with 2 μg of BmooF or BmooM venom, pooled venom (positive control) or PBS (negative control) diluted in 50 mM bicarbonate-carbonate buffer pH 9.6 (final volume of 100 μL per well). After an overnight incubation at 4 °C, the wells were washed three times with PBS pH 7.2, and further blocked with 250 μL of 2% skim milk in PBS (MPBS) for 2 h, at 37 °C. The wells were washed with PBS and incubated for 1 h, at 37 °C, with anti-bothropic serum diluted 1:1000 in 1% MPBS, at a final volume of 100 μL per well. Then, the wells were washed three times with PBS and 0.05% PBS-Tween, and further incubated with 100 μL of peroxidase-labelled anti-horse IgG antibodies diluted 1:3000 in 1% MPBS for 1 h, at room temperature. The wells were washed again as described above, before adding 100 μL of the OPD-H_2_O_2_ substrate solution (0.5 mg/mL *o*-phenylenediamine in 0.1 M citrate-phosphate buffer pH 5.0 and 0.03% hydrogen peroxide). After a 15-min incubation, when it was possible to detected color in the plate protected from light, the reaction was stopped with 50 μL of 1 M H_2_SO_4_ and absorbance was recorded at 490 nm in a microplate reader (Sunrise Tecan, Männedorf, Switzerland). Results were presented as relative percentage of serum recognition, in which pool venom immunoreactivity was considered 100% of recognition by the anti-bothropic serum. 

### 4.6. Statistical Analyses

Experimental data are presented as mean ± SD, and they were analysed with the aid of the GraphPad Prism software, version 6.0 for Windows (GraphPad Software, La Jolla, CA, USA, 2012). Data from enzyme activity were analysed using the Student’s *t-*test, while data from ELISA and cytotoxicity assays were analysed using one-way analysis of variance (ANOVA) followed by the Tukey’s test. Values of *p* < 0.05 were considered statistically significant. 

### 4.7. Fractionation of Crude Venom by Reversed-Phase High-Performance Liquid Chromatography (HPLC)

Approximately 5 mg of BmooF or BmooM venom was diluted in 500 µL of 0.1% trifluoroacetic acid (TFA) and applied onto a C18 reversed-phase HPLC column (0.46 × 25 cm; Shimadzu, Kyoto, Japan), previously equilibrated with 0.1% TFA (*v*/*v*). Elution was performed under the conditions reported by Bernardes and collaborators [55]: flow rate of 1 mL/min; linear concentration gradient of 70% acetonitrile (A) and 0.1% TFA (B) (5% B for 5 min, 5–15% B over 10 min, 15–45% B over 60 min, 45–70% B over 12 min). Protein elution was monitored by recording absorbance at 280 nm. The fractions were collected manually, pooled, lyophilized and stored at −20 °C for further characterization.

### 4.8. Proteomics Analyses

#### 4.8.1. Matrix Assisted Laser Desorption Ionization-Time of Flight (*MALDI*-TOF) Mass Spectrometry

Venom fractions whose chromatographic profiles markedly differed between the snake genders were further analysed by MALDI-TOF mass spectrometry. Briefly, aliquots of venom fractions suspended in water were desalted on 10-µL ZipTip^®^ pipette tips with C18 reversed-phase resin, using an acetonitrile/water/formic acid (49.8/50/0.2) solution as eluent. Next, the samples were spotted with 1 µL of 2,5-dihydroxybenzoic acid matrix (10 mg/mL in 0.2% formic acid and 50% acetonitrile). The samples were analysed in a MALDI-TOF UltrafleXtreme mass spectrometer (Bruker Daltonics, Bremen, Germany) operating in the reflected positive mode, equipped with a SmartBeam Laser in a mass range of 700–4000 *m*/*z*. The analyser was previously calibrated with Peptide Calibration Standard II. For each fraction, we accumulated at least 5000 records of mass spectra. The mass spectra were processed and analysed using DataAnalysis 4.0 software (Bruker Daltonics, Bremen, Germany, 2010). We selected the ions peaks with relative intensity over 5% and those ions with a higher resolution of the monoisotopic pattern (reflection mode). 

#### 4.8.2. Analysis in the AmaZon Speed ETD Ion Trap Mass Spectrometer

BmooM and BmooF venom samples (40 µg) were added to Laemmli buffer and heated at 100 °C, for 5 min. Proteins were separated on a 4–12% NuPAGE MES gel ran at 120 V, using SeeBlue Plus2™ pre-stained standard as molecular marker. After staining with Coomassie Brilliant Blue G-250, the gels were cut into three slices per band and submitted to in-gel trypsin digestion in the robot JANUS^®^ G3 Automated Workstations (Perkin-Elmer, Waltham, MA, USA), according to standard procedures. The resulting peptides (2 μL) were mixed with 9 μL of a solution containing 0.1% formic acid and 50% acetonitrile. The samples were injected into the UPLC M-Class system hyphenated to the AmaZon Speed ETD Ion Trap mass spectrometer (Bruker Daltonics, Bremen, Germany), with a total run time of 60 min (30-min gradient). The solution containing peptides was ionized by electrospray, and triply charged peptides were subjected to fragmentation by electron transfer dissociation (ETD). Mass spectra were analysed using the ProteinScape 3 software with the “Snake Venom” package from UniProt database downloaded in June 2017.

#### 4.8.3. Shotgun Proteomics Using the Q-Exactive Orbitrap Mass Spectrometer

BmooM and BmooF venom samples (20 µg) were suspended in 159 µL of 50 mM NH_4_HCO_3_ pH 7.8 and reduced with 3.2 µL of 500 mM dithiothreitol for 40 min, at 56 °C, under shaking at 300 rpm. Next, the samples were alkylated with 6.4 µL of 500 mM iodoacetamide for 30 min, at room temperature, in the dark, and reduced with 3.6 µL of 500 mM dithiothreitol for further 30 min, at room temperature. Then, the venom samples were submitted to two trypsin digestions in 50 mM NH_4_HCO_3_ pH 7.8: in the first one, the protein:trypsin mixture at a ratio of 1:50 was incubated overnight, at 37 °C, under shaking at 300 rpm; in the second one, the protein:trypsin mixture at a ratio of 1:100 supplemented with 640 µL of 100% acetonitrile was incubated for 3 h, at 37 °C, under shaking at 300 rpm. Reactions were stopped by adding 10% TFA to the reaction mixtures, and the samples were dried on speed vacuum. 

For shotgun proteomics analysis, the samples were suspended in 20 µL of 0.1% TFA for desalting on ZipTip™ pipette tips with C18 resin, using an acetonitrile/water/TFA (49.8/50/0.2 *v*/*v*) solution as eluent. The digested material was analysed in the Acquity UPLC^®^ M-Class (Waters, Milford, MA, USA) coupled to the Q-Exactive™ Plus Hybrid Quadrupole-Orbitrap™ Mass Spectrometer (Thermo Scientific, Bremen, Germany). Peptides were eluted using a gradient of 2–90% of solution B in 150 min (A: water/0.1% formic acid; B: acetonitrile), at a flow rate of 0.6 mL/min, and data were acquired in the positive-ion mode.

Protein identification by automated de novo sequencing was performed with Peaks Studio 7.0 software [56], with “Snake Venom” package from UniProt database downloaded in January 2018 (33,138 sequences), and the *B. moojeni* transcriptome data deposited by Amorim et al. (2017) (Transcriptome Shotgun Assembly accession GFWW00000000). Carbamidomethylation was set as fixed modification, while oxidation (M) was set as variable modifications, with maximum mixed cleavages at 3. Parent mass and fragment mass error tolerance were set at 5 ppm and 0.015 Da, respectively. False discovery rate (FDR) of 1% and unique peptide ≥2 were used for filtering out inaccurate proteins for the SPIDER search. A −10lgP > 20 indicates that the detected proteins are relatively high in confidence as it targets very few decoy matches above that threshold. 

The percentage of the venom protein family in each crude venom was calculated as described by Abidin and colleagues [41], using the following formula:

$$\frac{\mathrm{number}\;\mathrm{of}\;\mathrm{proteins}\;\left(\mathrm{protein}\;\mathrm{family}\right)}{\mathrm{total}\;\mathrm{proteins}\;\mathrm{detected}\;\mathrm{using}\;\mathrm{LC}-\mathrm{MS}/\mathrm{MS}}\times 100$$

## Figures and Tables

**Table 1:** Full-length transcripts identified in *B. moojeni* venom proteome from *B. moojeni* transcriptome.

Accession	−10lgP	Coverage (%)	Number of Peptides	Number of Unique Peptides	Average Mass	Description
ATU85535.1	489.98	93	316	316	56,840	L-amino acid oxidase
ATU85523.1	426.4	94	164	164	24,669	Snake venom metalloproteinase BmooMPalpha-I-like isoform
ATU85528.1	376.13	88	71	71	15,699	Basic phospholipase A2 myotoxin
ATU85541.1	334.57	85	35	35	26,837	Cysteine-rich secretory protein Moojin
ATU85526.1	322.31	54	43	43	63,875	Phospholipase B
ATU85531.1	272.92	61	23	23	16,334	Snake venom vascular endothelial growth factor toxin
ATU85542.1	172.96	16	7	7	52,550	Hyaluronidase BmooHyal-1
ATU85551.1	172.41	38	4	4	9767	Waprin 1
ATU85533.1	109.82	30	5	4	16,657	C-type lectin isoform 1

**Table 2:** Results statistics of venom from male (BmooM) and female (BmooF) *B. moojeni* snakes by mass spectrometry followed by analysis against Uniprot database.

**Statistics Data**		
	**BmooM**	**BmooF**
Number of MS scans	6676	6586
Number of MS/MS scans	42,843	39,953
**Statistics of filtered result**		
Peptide-Spectrum Matches	12,453	9421
Peptide Sequences	3148	3349
Protein Groups	142	206
Proteins	191	252
Proteins (number of Unique Peptides)	103 (>2); 88 (=2); 0 (=1)	149 (>2); 103 (=2); 0 (=1)
*De novo* Only Spectra	1445	1040

## Supplementary Materials

The following are available online at http://www.mdpi.com/2072-6651/10/5/177/s1, Table S1: Full list of proteins identified after proteomics analysis of pooled *Bothrops moojeni* venom samples against the database from the transcriptome of *B. moojeni* venom gland. Table S2: Full list of peptides identified after proteomics analysis of pooled *Bothrops moojeni* venom samples analysed against the “Snake Venom” package from UniProt database. Table S3: Parameters of the peptides found exclusively in venom from male *B. moojeni* snakes (BmooM). Table S4: Parameters of the peptides found exclusively in venom from female *B. moojeni* snakes (BmooF).
